# Supplementary material for: Rutin/Sulfobutylether-β-Cyclodextrin as a Promising Therapeutic Formulation for Ocular Infection
Source: Pharmaceutics. 2024 Feb 5;16(2):233. doi: 10.3390/pharmaceutics16020233 (PMC10892075; doi:10.3390/pharmaceutics16020233)
Supplement: Supplementary file 1 [file pharmaceutics-16-00233-s001.zip › pharmaceutics-2844843-supplementary.pdf]

pharmaceutics

MDPI

Supporting Information

Rutin/sulfobutylether-β-cyclodextrin as a promising therapeutic agent/formulation for ocular infection

Federica De Gaetano [1], Martina Pastorello [1], Venerando Pistarà [2], Antonio Rescifina [2], Fatima Margani [3], Vincenzina Barbera [3], Cinzia Anna Ventura [1,*] and Andreana Marino [1,*]

[1] Dipartimento di Scienze Chimiche, Biologiche, Farmaceutiche e Ambientali, Università di Messina, Viale Ferdinando Stagno d'Alcontres 31, 98166 Messina, Italy; fedegaetano@unime.it (F.D.G.); martina.pastorello@studenti.unime.it (M.P.);
[2] Dipartimento di Scienze del Farmaco e della Salute, Università di Catania, Viale A. Doria 6, 95125 Catania, Italy; vpistara@unict.it (V.P.); arescifina@unict.it (A.R.);
[3] Dipartimento di Chimica, Materiali e Ingegneria Chimica "G. Natta", Politecnico di Milano, Via Mancinelli 7, 20131 Milano, Italy; fatima.margani@polimi.it (F.M.); vincenzina.barbera@polimi.it (V.B.).
* Correspondence: caventura@unime.it (C.A.V.); anmarino@unime.it (A.M.)

Initial deployment of RTN for MD simulations

To start the MD simulation with a plausible arrangement of host and guest molecules, we conducted a molecular docking experiment employing the AutoDock 4.2.6 software implemented in YASARA.

The maps were generated by the program AutoGrid (4.2.6) with a spacing of 0.375 Å and dimensions that encompass all atoms extending 10 Å from the surface of the AM1 minimized structure of the SBE-β-CD. All the parameters were inserted at default settings. In the docking tab, the macromolecule and ligand are selected, and GA parameters are set as ga_runs = 100, ga_pop_size = 150, ga_num_evals = 20000000, ga_num_generations = 27000, ga_elitism = 1, ga_mutation_rate = 0.02, ga_crossover_rate = 0.8, ga_crossover_mode = two points, ga_cauchy_alpha = 0.0, ga_cauchy_beta = 1.0, number of generations for picking worst individual = 10.

The results of docking indicate that between the best poses of RTN, there is one in which it is inserted in the SBE-β-CD cavity from the upper rim with the ring X inside the cavity and the other one in which RTN is inserted in the SBE-β-CD cavity from the upper rim with the ring Y inside the cavity.

Free binding energy calculation

A well-known and widely used molecular mechanics/Poisson-Boltzmann surface area (MM/PBSA) approach was employed to estimate the binding free energy that can be achieved from the difference between the free energies of the RTN/SBE-*β*-CD complex ($\Delta G_{complex}$), free SBE-*β*-CD (ΔG_{CD}) and RTN (ΔG_{RTN}) according to the following equation:

$$\Delta G_{bind} = \Delta G_{complex} - (\Delta G_{CD} + \Delta G_{RTN}) \qquad \text{eq. (S1)}$$

The total free energy of each molecule typically contains the enthalpy (ΔH) and entropy (ΔS) contributions, as given in Equation (S2):

$$\Delta G = \Delta H - T\Delta S \qquad \text{eq. (S2)}$$

where the ΔH of any system is composed of the enthalpy changes in the gas phase upon complex formation (ΔE_{MM}) and the solvated free energy contribution (ΔG_{solv}), while the $T\Delta S$ is the change of conformational entropy upon the complex formation.

Therefore, Equation (S2) can be re-written as:

$$\Delta G = \Delta E_{MM} + \Delta G_{sol} - T\Delta S \qquad \text{eq. (S3)}$$

where ΔE_{MM} is the summation of the bonded (ΔG_{bonded}) and nonbonded van der Waals (ΔE_{vdW}) and electrostatic (ΔE_{ele}) energies evaluated with YASARA software.

$$\Delta E_{MM} = \Delta E_{bonnded} + \Delta E_{vdW} + \Delta E_{MM} \quad \text{eq. (S4)}$$

The solvation free energy (ΔG_{solv}) is divided into the polar (ΔG_{psolv}) and non-polar (ΔG_{npsolv}) terms as shown in Equation (S5).

$$\Delta G_{solv} = \Delta G_{psolv} + \Delta G_{nsolv} \quad \text{eq. (S5)}$$

The polar solvation free energy component was computed by the Poisson–Boltzmann (PB) equation, while the non-polar term was estimated from a linear relation according to Equation (6):

$\Delta G_{nsolv} =$ [illegible]SASA + [illegible] eq. (S6)

where SASA is the solvent accessible surface area of each given molecule and is determined using a solvent probe radius of 1.4 Å. The values of the surface tension γ and the offset β were set to 0.00542 kcal/molÅ^2 and 0.92 kcal/mol, respectively. Dielectric constants of 1.0 and 80.0 were used for the solute and solvent, respectively. The entropy term (ΔS) consisting of translational, rotational and vibrational contributions was determined by normal mode analysis.
